# Crystal structure of 2-butyl­sulfanyl-4,6-bis­[(*E*)-styr­yl]pyrimidine

**DOI:** 10.1107/S2056989015008166

**Published:** 2015-04-30

**Authors:** Aijian Wang, Guanghui Li

**Affiliations:** a China-Australia Joint Research Center for Functional Molecular Materials, Scientific Research Academy & School of Chemistry and Chemical Engineering, Jiangsu University, Zhenjiang 212013, People’s Republic of China

**Keywords:** crystal structure, weak inter­actions, pyrimidine

## Abstract

In the title compound, C_24_H_24_N_2_S, the dihedral angles between the central pyrimidine ring and pendant benzene rings are 18.46 (6) and 5.95 (6)°. The butyl­sulfanyl side chain adopts a twisted conformation [S—C—C—C = 177.34 (10)° and C—C—C—C = 67.68 (18)°]. No directional inter­actions beyond typical van der Waals contacts could be identified in the crystal.

## Related literature   

For general background to pyrimidine derivatives and their applications, see: Walker *et al.* (2009 ▸); van Laar *et al.* (2001 ▸); Casas *et al.* (2006 ▸); Deng *et al.* (2008 ▸); Nguyen (2008 ▸). For the synthesis of the title compound, see: Liu *et al.* (2007 ▸).
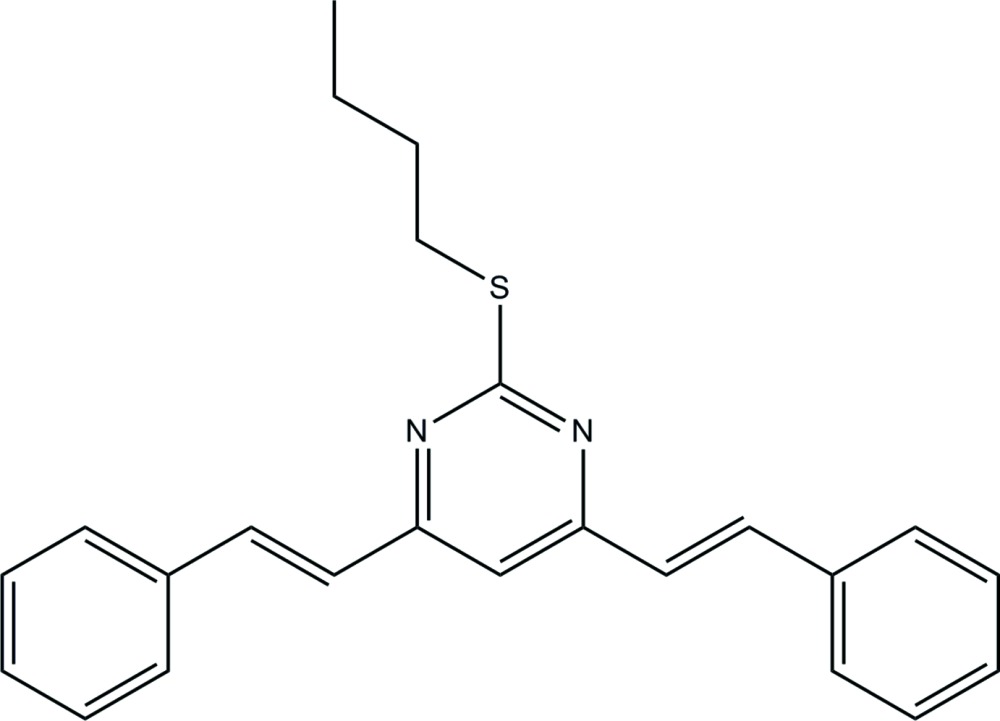


## Experimental   

### Crystal data  

C_24_H_24_N_2_S
*M* *_r_* = 372.51
Monoclinic, 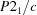

*a* = 9.0447 (18) Å
*b* = 9.3798 (19) Å
*c* = 23.802 (7) Å
β = 103.44 (3)°
*V* = 1964.0 (8) Å^3^
*Z* = 4
Mo *K*α radiation
μ = 0.18 mm^−1^
*T* = 153 K
0.26 × 0.23 × 0.22 mm

### Data collection  

Rigaku Saturn 724+ CCD diffractometer
Absorption correction: multi-scan multi-scan *T*_min_ = 0.830, *T*_max_ = 1.000
9602 measured reflections
3487 independent reflections
3193 reflections with *I* > 2σ(*I*)
*R*_int_ = 0.020

### Refinement  

*R*[*F* ^2^ > 2σ(*F* ^2^)] = 0.034
*wR*(*F* ^2^) = 0.087
*S* = 1.06
3487 reflections
245 parameters
H-atom parameters constrained
Δρ_max_ = 0.20 e Å^−3^
Δρ_min_ = −0.23 e Å^−3^

### 

Data collection: *CrystalClear* (Rigaku, 2008 ▸); cell refinement: *CrystalClear*; data reduction: *CrystalClear*; program(s) used to solve structure: *SHELXTL* (Sheldrick, 2008 ▸); program(s) used to refine structure: *SHELXTL*; molecular graphics: *SHELXTL*; software used to prepare material for publication: *SHELXTL*.

## Supplementary Material

Crystal structure: contains datablock(s) I, New_Global_Publ_Block. DOI: 10.1107/S2056989015008166/hb7413sup1.cif

Structure factors: contains datablock(s) I. DOI: 10.1107/S2056989015008166/hb7413Isup2.hkl

Supporting information file. DOI: 10.1107/S2056989015008166/hb7413Isup3.cml

Supporting information file. DOI: 10.1107/S2056989015008166/hb7413Isup4.docx

**Fig. 1.**
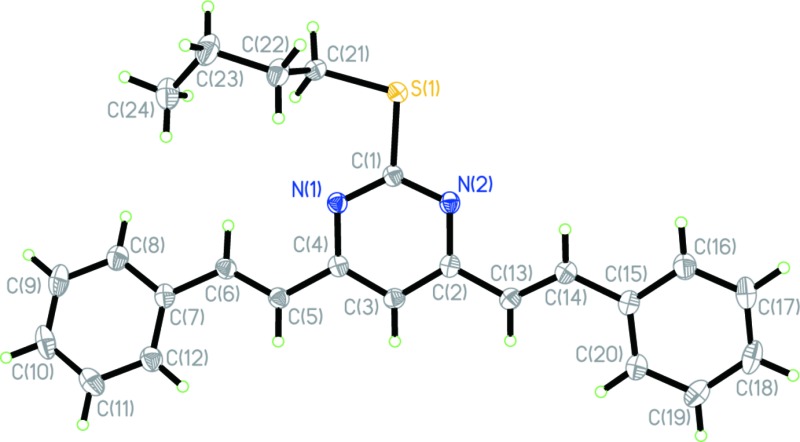
The molecular structure of (I) showing 50% displacement ellipsoids.

. DOI: 10.1107/S2056989015008166/hb7413fig1.tif
The mol­ecular structure of (I) showing 50% displacement ellipsoids.

**Fig. 2.**
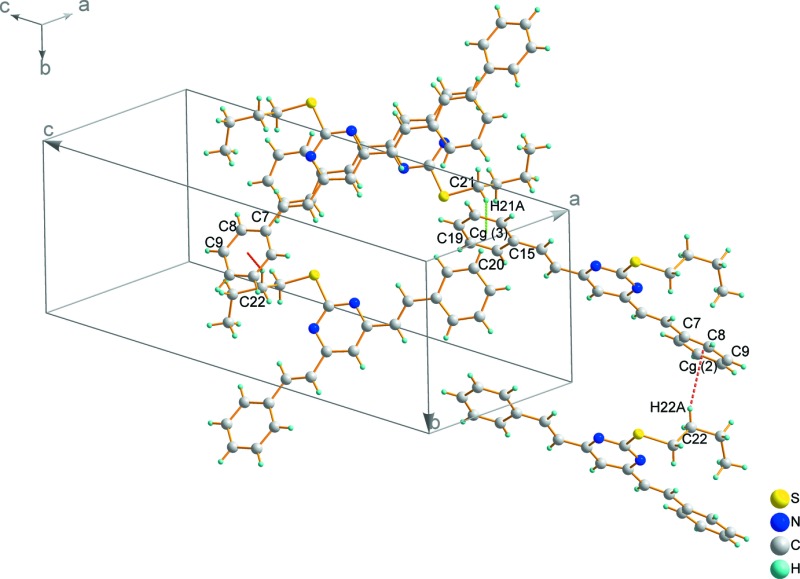
Packing diagram for (I).

. DOI: 10.1107/S2056989015008166/hb7413fig2.tif
Packing diagram for (I).

CCDC reference: 1010472

Additional supporting information:  crystallographic information; 3D view; checkCIF report

## supplementary crystallographic information

### Crystal data

**Table:**

C_24_H_24_N_2_S	*F*(000) = 792
*M**_r_* = 372.51	*D*_x_ = 1.260 Mg m^−^^3^
Monoclinic, *P*2_1_/*c*	Mo *K*α radiation, λ = 0.71073 Å
*a* = 9.0447 (18) Å	Cell parameters from 4606 reflections
*b* = 9.3798 (19) Å	θ = 3.9–28.6°
*c* = 23.802 (7) Å	µ = 0.18 mm^−^^1^
β = 103.44 (3)°	*T* = 153 K
*V* = 1964.0 (8) Å^3^	Prism, yellow
*Z* = 4	0.26 × 0.23 × 0.22 mm

### Data collection

**Table:**

Rigaku CCD diffractometer	3487 independent reflections
Radiation source: fine-focus sealed tube	3193 reflections with *I* > 2σ(*I*)
Graphite monochromator	*R*_int_ = 0.020
phi and ω scans	θ_max_ = 25.2°, θ_min_ = 3.9°
Absorption correction: multi-scan multi-scan	*h* = −10→8
*T*_min_ = 0.830, *T*_max_ = 1.000	*k* = −10→11
9602 measured reflections	*l* = −28→28

### Refinement

**Table:**

Refinement on *F*^2^	Primary atom site location: structure-invariant direct methods
Least-squares matrix: full	Secondary atom site location: difference Fourier map
*R*[*F*^2^ > 2σ(*F*^2^)] = 0.034	Hydrogen site location: inferred from neighbouring sites
*wR*(*F*^2^) = 0.087	H-atom parameters constrained
*S* = 1.06	*w* = 1/[σ^2^(*F*_o_^2^) + (0.0468*P*)^2^ + 0.4259*P*] where *P* = (*F*_o_^2^ + 2*F*_c_^2^)/3
3487 reflections	(Δ/σ)_max_ = 0.002
245 parameters	Δρ_max_ = 0.20 e Å^−^^3^
0 restraints	Δρ_min_ = −0.23 e Å^−^^3^

### Special details

**Table:**

Geometry. All e.s.d.'s (except the e.s.d. in the dihedral angle between two l.s. planes) are estimated using the full covariance matrix. The cell e.s.d.'s are taken into account individually in the estimation of e.s.d.'s in distances, angles and torsion angles; correlations between e.s.d.'s in cell parameters are only used when they are defined by crystal symmetry. An approximate (isotropic) treatment of cell e.s.d.'s is used for estimating e.s.d.'s involving l.s. planes.
Refinement. Refinement of *F*^2^ against ALL reflections. The weighted *R*-factor *wR* and goodness of fit *S* are based on *F*^2^, conventional *R*-factors *R* are based on *F*, with *F* set to zero for negative *F*^2^. The threshold expression of *F*^2^ > σ(*F*^2^) is used only for calculating *R*-factors(gt) *etc*. and is not relevant to the choice of reflections for refinement. *R*-factors based on *F*^2^ are statistically about twice as large as those based on *F*, and *R*- factors based on ALL data will be even larger.

### Fractional atomic coordinates and isotropic or equivalent isotropic displacement parameters (Å^2^)

**Table:**

	*x*	*y*	*z*	*U*_iso_*/*U*_eq_	
S1	0.14918 (4)	0.26493 (4)	0.884447 (14)	0.02603 (12)	
N1	0.29497 (11)	0.49599 (11)	0.93583 (4)	0.0198 (2)	
N2	0.14298 (11)	0.35823 (11)	0.98633 (4)	0.0202 (2)	
C1	0.20397 (13)	0.38866 (13)	0.94166 (5)	0.0193 (3)	
C2	0.17542 (13)	0.45128 (13)	1.03058 (5)	0.0194 (3)	
C3	0.26563 (13)	0.57051 (13)	1.02829 (5)	0.0205 (3)	
H3	0.2852	0.6377	1.0590	0.025*	
C4	0.32650 (13)	0.58934 (13)	0.98031 (5)	0.0191 (3)	
C5	0.42814 (13)	0.70891 (13)	0.97653 (5)	0.0204 (3)	
H5	0.4634	0.7661	1.0099	0.025*	
C6	0.47367 (14)	0.74153 (13)	0.92880 (6)	0.0219 (3)	
H6	0.4330	0.6851	0.8957	0.026*	
C7	0.57954 (14)	0.85485 (13)	0.92161 (6)	0.0225 (3)	
C8	0.60175 (15)	0.88109 (15)	0.86649 (6)	0.0281 (3)	
H8	0.5451	0.8285	0.8346	0.034*	
C9	0.70538 (16)	0.98295 (16)	0.85754 (7)	0.0343 (4)	
H9	0.7194	0.9993	0.8197	0.041*	
C10	0.78780 (16)	1.06029 (15)	0.90348 (7)	0.0353 (4)	
H10	0.8589	1.1298	0.8974	0.042*	
C11	0.76658 (16)	1.03641 (15)	0.95844 (7)	0.0332 (3)	
H11	0.8230	1.0901	0.9901	0.040*	
C12	0.66348 (15)	0.93468 (14)	0.96766 (6)	0.0273 (3)	
H12	0.6498	0.9192	1.0056	0.033*	
C13	0.11375 (13)	0.42439 (14)	1.08114 (5)	0.0210 (3)	
H13	0.1239	0.4973	1.1095	0.025*	
C14	0.04425 (14)	0.30423 (14)	1.08999 (5)	0.0222 (3)	
H14	0.0362	0.2324	1.0613	0.027*	
C15	−0.02107 (14)	0.27106 (14)	1.13927 (5)	0.0218 (3)	
C16	−0.09987 (15)	0.14320 (15)	1.13976 (6)	0.0286 (3)	
H16	−0.1076	0.0781	1.1086	0.034*	
C17	−0.16687 (16)	0.10953 (16)	1.18474 (7)	0.0341 (3)	
H17	−0.2208	0.0224	1.1841	0.041*	
C18	−0.15537 (16)	0.20253 (17)	1.23059 (6)	0.0327 (3)	
H18	−0.2010	0.1794	1.2616	0.039*	
C19	−0.07708 (15)	0.32952 (16)	1.23120 (6)	0.0302 (3)	
H19	−0.0692	0.3936	1.2627	0.036*	
C20	−0.01020 (15)	0.36359 (15)	1.18626 (6)	0.0265 (3)	
H20	0.0438	0.4508	1.1873	0.032*	
C21	0.25447 (15)	0.32567 (15)	0.83313 (5)	0.0252 (3)	
H21A	0.2058	0.2874	0.7945	0.030*	
H21B	0.2489	0.4310	0.8308	0.030*	
C22	0.42011 (15)	0.28109 (15)	0.84837 (6)	0.0286 (3)	
H22A	0.4260	0.1761	0.8524	0.034*	
H22B	0.4702	0.3232	0.8861	0.034*	
C23	0.50566 (18)	0.32712 (16)	0.80329 (7)	0.0370 (4)	
H23A	0.6071	0.2814	0.8124	0.044*	
H23B	0.4501	0.2916	0.7650	0.044*	
C24	0.52647 (17)	0.48708 (16)	0.79936 (7)	0.0364 (4)	
H24A	0.5838	0.5078	0.7701	0.055*	
H24B	0.5823	0.5235	0.8369	0.055*	
H24C	0.4267	0.5333	0.7886	0.055*	

### Atomic displacement parameters (Å^2^)

**Table:**

	*U*^11^	*U*^22^	*U*^33^	*U*^12^	*U*^13^	*U*^23^
S1	0.0269 (2)	0.0267 (2)	0.0270 (2)	−0.00895 (13)	0.01143 (15)	−0.00718 (14)
N1	0.0190 (5)	0.0189 (6)	0.0221 (5)	0.0003 (4)	0.0062 (4)	0.0002 (4)
N2	0.0189 (5)	0.0199 (6)	0.0226 (5)	0.0010 (4)	0.0066 (4)	0.0013 (4)
C1	0.0174 (6)	0.0188 (6)	0.0219 (6)	0.0015 (5)	0.0051 (5)	0.0001 (5)
C2	0.0164 (6)	0.0205 (7)	0.0214 (6)	0.0038 (5)	0.0045 (5)	0.0033 (5)
C3	0.0208 (6)	0.0202 (7)	0.0206 (6)	0.0011 (5)	0.0048 (5)	−0.0007 (5)
C4	0.0169 (6)	0.0185 (6)	0.0216 (6)	0.0028 (5)	0.0042 (5)	0.0015 (5)
C5	0.0203 (6)	0.0180 (6)	0.0231 (6)	−0.0003 (5)	0.0052 (5)	−0.0008 (5)
C6	0.0230 (7)	0.0187 (6)	0.0248 (7)	−0.0007 (5)	0.0071 (5)	−0.0010 (5)
C7	0.0218 (6)	0.0184 (6)	0.0292 (7)	0.0026 (5)	0.0096 (5)	0.0023 (5)
C8	0.0299 (7)	0.0268 (7)	0.0299 (7)	0.0007 (6)	0.0116 (6)	0.0030 (6)
C9	0.0355 (8)	0.0322 (8)	0.0410 (9)	0.0035 (6)	0.0207 (7)	0.0129 (7)
C10	0.0260 (7)	0.0243 (8)	0.0595 (10)	−0.0016 (6)	0.0178 (7)	0.0089 (7)
C11	0.0267 (7)	0.0249 (7)	0.0471 (9)	−0.0038 (6)	0.0069 (7)	−0.0020 (7)
C12	0.0270 (7)	0.0235 (7)	0.0327 (7)	−0.0010 (5)	0.0094 (6)	0.0005 (6)
C13	0.0195 (6)	0.0226 (7)	0.0210 (6)	0.0024 (5)	0.0051 (5)	0.0007 (5)
C14	0.0226 (6)	0.0226 (7)	0.0217 (6)	0.0023 (5)	0.0058 (5)	0.0013 (5)
C15	0.0186 (6)	0.0241 (7)	0.0231 (7)	0.0038 (5)	0.0056 (5)	0.0048 (5)
C16	0.0299 (7)	0.0266 (7)	0.0306 (7)	−0.0012 (6)	0.0099 (6)	0.0026 (6)
C17	0.0311 (8)	0.0331 (8)	0.0411 (8)	−0.0036 (6)	0.0148 (7)	0.0108 (7)
C18	0.0291 (8)	0.0413 (9)	0.0324 (8)	0.0077 (6)	0.0171 (6)	0.0142 (7)
C19	0.0322 (7)	0.0361 (8)	0.0240 (7)	0.0080 (6)	0.0102 (6)	0.0040 (6)
C20	0.0277 (7)	0.0271 (7)	0.0261 (7)	0.0008 (6)	0.0092 (6)	0.0043 (6)
C21	0.0269 (7)	0.0296 (7)	0.0195 (6)	−0.0036 (6)	0.0066 (5)	−0.0028 (6)
C22	0.0297 (7)	0.0272 (7)	0.0324 (8)	0.0029 (6)	0.0146 (6)	0.0036 (6)
C23	0.0427 (9)	0.0314 (8)	0.0454 (9)	0.0054 (7)	0.0276 (7)	0.0035 (7)
C24	0.0350 (8)	0.0343 (8)	0.0436 (9)	−0.0003 (6)	0.0171 (7)	0.0063 (7)

### Geometric parameters (Å, º)

**Table:**

S1—C1	1.7701 (13)	C13—H13	0.9500
S1—C21	1.8066 (14)	C14—C15	1.4642 (18)
N1—C1	1.3279 (16)	C14—H14	0.9500
N1—C4	1.3523 (16)	C15—C16	1.3966 (19)
N2—C1	1.3375 (16)	C15—C20	1.4009 (19)
N2—C2	1.3466 (16)	C16—C17	1.384 (2)
C2—C3	1.3929 (18)	C16—H16	0.9500
C2—C13	1.4611 (18)	C17—C18	1.382 (2)
C3—C4	1.3896 (17)	C17—H17	0.9500
C3—H3	0.9500	C18—C19	1.384 (2)
C4—C5	1.4662 (17)	C18—H18	0.9500
C5—C6	1.3309 (18)	C19—C20	1.3831 (19)
C5—H5	0.9500	C19—H19	0.9500
C6—C7	1.4670 (18)	C20—H20	0.9500
C6—H6	0.9500	C21—C22	1.5160 (19)
C7—C8	1.3946 (19)	C21—H21A	0.9900
C7—C12	1.3969 (19)	C21—H21B	0.9900
C8—C9	1.389 (2)	C22—C23	1.5239 (19)
C8—H8	0.9500	C22—H22A	0.9900
C9—C10	1.378 (2)	C22—H22B	0.9900
C9—H9	0.9500	C23—C24	1.518 (2)
C10—C11	1.384 (2)	C23—H23A	0.9900
C10—H10	0.9500	C23—H23B	0.9900
C11—C12	1.3868 (19)	C24—H24A	0.9800
C11—H11	0.9500	C24—H24B	0.9800
C12—H12	0.9500	C24—H24C	0.9800
C13—C14	1.3310 (19)		
			
C1—S1—C21	102.48 (6)	C15—C14—H14	116.6
C1—N1—C4	115.60 (11)	C16—C15—C20	117.82 (12)
C1—N2—C2	115.35 (11)	C16—C15—C14	119.39 (12)
N1—C1—N2	128.69 (12)	C20—C15—C14	122.78 (12)
N1—C1—S1	119.09 (9)	C17—C16—C15	121.23 (13)
N2—C1—S1	112.22 (9)	C17—C16—H16	119.4
N2—C2—C3	120.87 (11)	C15—C16—H16	119.4
N2—C2—C13	118.47 (11)	C18—C17—C16	120.05 (14)
C3—C2—C13	120.66 (12)	C18—C17—H17	120.0
C4—C3—C2	118.80 (12)	C16—C17—H17	120.0
C4—C3—H3	120.6	C17—C18—C19	119.73 (13)
C2—C3—H3	120.6	C17—C18—H18	120.1
N1—C4—C3	120.63 (11)	C19—C18—H18	120.1
N1—C4—C5	117.93 (11)	C20—C19—C18	120.36 (14)
C3—C4—C5	121.43 (12)	C20—C19—H19	119.8
C6—C5—C4	123.44 (12)	C18—C19—H19	119.8
C6—C5—H5	118.3	C19—C20—C15	120.80 (13)
C4—C5—H5	118.3	C19—C20—H20	119.6
C5—C6—C7	127.30 (12)	C15—C20—H20	119.6
C5—C6—H6	116.4	C22—C21—S1	113.46 (9)
C7—C6—H6	116.4	C22—C21—H21A	108.9
C8—C7—C12	118.20 (12)	S1—C21—H21A	108.9
C8—C7—C6	118.57 (12)	C22—C21—H21B	108.9
C12—C7—C6	123.19 (12)	S1—C21—H21B	108.9
C9—C8—C7	121.04 (14)	H21A—C21—H21B	107.7
C9—C8—H8	119.5	C21—C22—C23	112.88 (12)
C7—C8—H8	119.5	C21—C22—H22A	109.0
C10—C9—C8	120.01 (14)	C23—C22—H22A	109.0
C10—C9—H9	120.0	C21—C22—H22B	109.0
C8—C9—H9	120.0	C23—C22—H22B	109.0
C9—C10—C11	119.78 (13)	H22A—C22—H22B	107.8
C9—C10—H10	120.1	C24—C23—C22	114.46 (12)
C11—C10—H10	120.1	C24—C23—H23A	108.6
C10—C11—C12	120.47 (14)	C22—C23—H23A	108.6
C10—C11—H11	119.8	C24—C23—H23B	108.6
C12—C11—H11	119.8	C22—C23—H23B	108.6
C11—C12—C7	120.50 (13)	H23A—C23—H23B	107.6
C11—C12—H12	119.8	C23—C24—H24A	109.5
C7—C12—H12	119.8	C23—C24—H24B	109.5
C14—C13—C2	124.15 (12)	H24A—C24—H24B	109.5
C14—C13—H13	117.9	C23—C24—H24C	109.5
C2—C13—H13	117.9	H24A—C24—H24C	109.5
C13—C14—C15	126.89 (13)	H24B—C24—H24C	109.5
C13—C14—H14	116.6		
			
C4—N1—C1—N2	−2.18 (18)	C8—C9—C10—C11	−0.2 (2)
C4—N1—C1—S1	177.30 (9)	C9—C10—C11—C12	0.3 (2)
C2—N2—C1—N1	1.89 (18)	C10—C11—C12—C7	0.0 (2)
C2—N2—C1—S1	−177.61 (8)	C8—C7—C12—C11	−0.42 (19)
C21—S1—C1—N1	3.44 (11)	C6—C7—C12—C11	177.28 (12)
C21—S1—C1—N2	−177.00 (9)	N2—C2—C13—C14	9.40 (18)
C1—N2—C2—C3	0.51 (16)	C3—C2—C13—C14	−170.62 (12)
C1—N2—C2—C13	−179.51 (10)	C2—C13—C14—C15	−179.42 (11)
N2—C2—C3—C4	−2.32 (17)	C13—C14—C15—C16	175.44 (12)
C13—C2—C3—C4	177.70 (11)	C13—C14—C15—C20	−3.6 (2)
C1—N1—C4—C3	0.06 (16)	C20—C15—C16—C17	0.83 (19)
C1—N1—C4—C5	179.18 (10)	C14—C15—C16—C17	−178.22 (12)
C2—C3—C4—N1	2.01 (17)	C15—C16—C17—C18	−0.6 (2)
C2—C3—C4—C5	−177.07 (11)	C16—C17—C18—C19	0.2 (2)
N1—C4—C5—C6	9.66 (18)	C17—C18—C19—C20	−0.1 (2)
C3—C4—C5—C6	−171.24 (12)	C18—C19—C20—C15	0.4 (2)
C4—C5—C6—C7	−177.30 (11)	C16—C15—C20—C19	−0.72 (18)
C5—C6—C7—C8	−173.89 (12)	C14—C15—C20—C19	178.30 (12)
C5—C6—C7—C12	8.4 (2)	C1—S1—C21—C22	78.82 (11)
C12—C7—C8—C9	0.54 (19)	S1—C21—C22—C23	177.34 (10)
C6—C7—C8—C9	−177.27 (12)	C21—C22—C23—C24	67.68 (18)
C7—C8—C9—C10	−0.2 (2)		
