# Supplementary material for: Crystal structure of 2-butyl­sulfanyl-4,6-bis­[(E)-styr­yl]pyrimidine
Source: Acta Crystallogr E Crystallogr Commun. 2015 Apr 30;71(Pt 5):o368. doi: 10.1107/S2056989015008166 (PMC4420047; doi:10.1107/S2056989015008166)

**Crystal structures of 2-(butylthio)-4,6-di((E)-styryl)pyrimidine**

**Aijian Wang* and Guanghui Li**

**S1** Synthesis and crystallization

2-(butylthio)-4,6-dimethylpyrimidine (2.95 g, 15 mmol) and benzaldehyde (3.5 g, 33 mmol) were added in an aqueous solution of sodium hydroxide (5 M, 50 ml) containing tetrabutylammonium iodide (10 mol % versus the heterocycle) and mixed. The mixture was heated under reflux for 3 h. After cooling, the reaction mixture was extracted with dichloromethane (120 ml × 3). The extract solution was dried with magnesium sulfate, and the solvent was removed by evaporation under reduced pressure. The crude product was recrystallized to afford the desired pale-yellow micro-crystals 1 (3.85 g, yield: 69%).

**Figure 1**

The molecular structure of (I) showing 50% displacement ellipsoids.


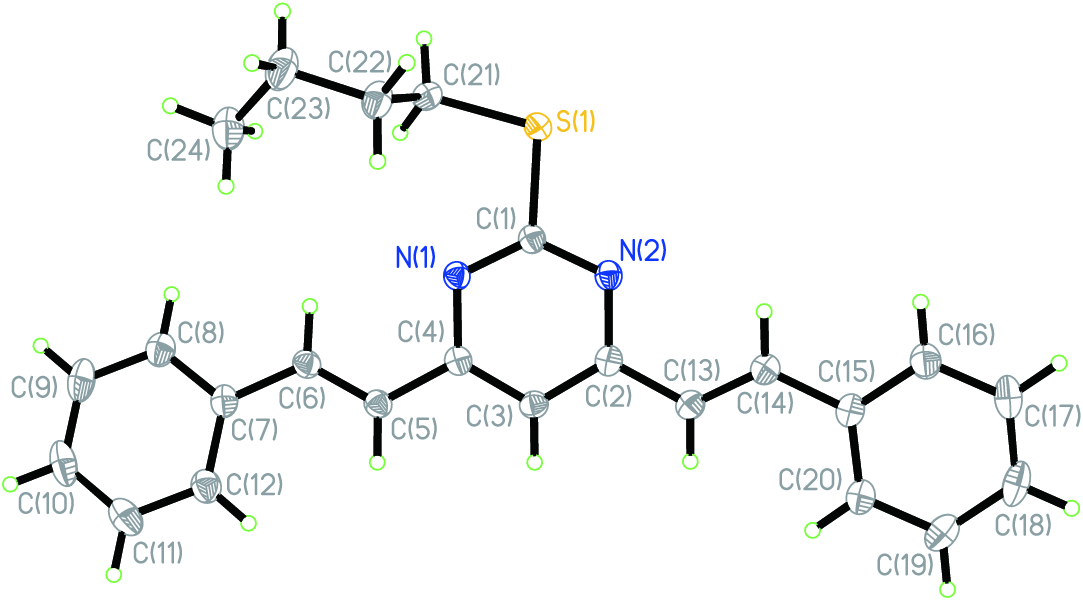


**Figure 2**

Packing diagram for (I).


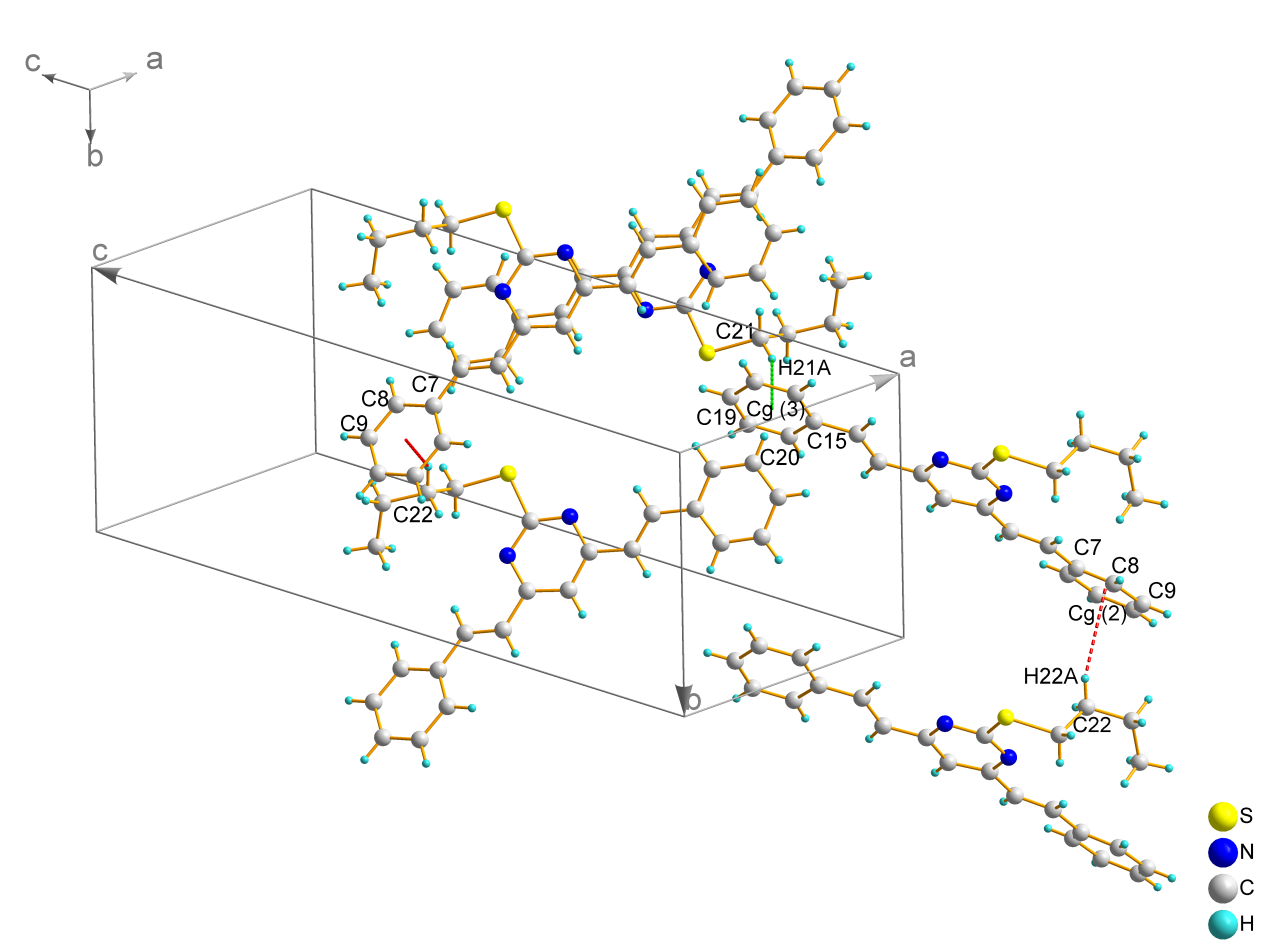

Supplement: Supplementary file 4 [file e-71-0o368-Isup4.docx]
