# Supplementary figures and images for: Crystal structure of 2-butyl­sulfanyl-4,6-bis­[(E)-styr­yl]pyrimidine
Source: Acta Crystallogr E Crystallogr Commun. 2015 Apr 30;71(Pt 5):o368. doi: 10.1107/S2056989015008166 (PMC4420047; doi:10.1107/S2056989015008166)

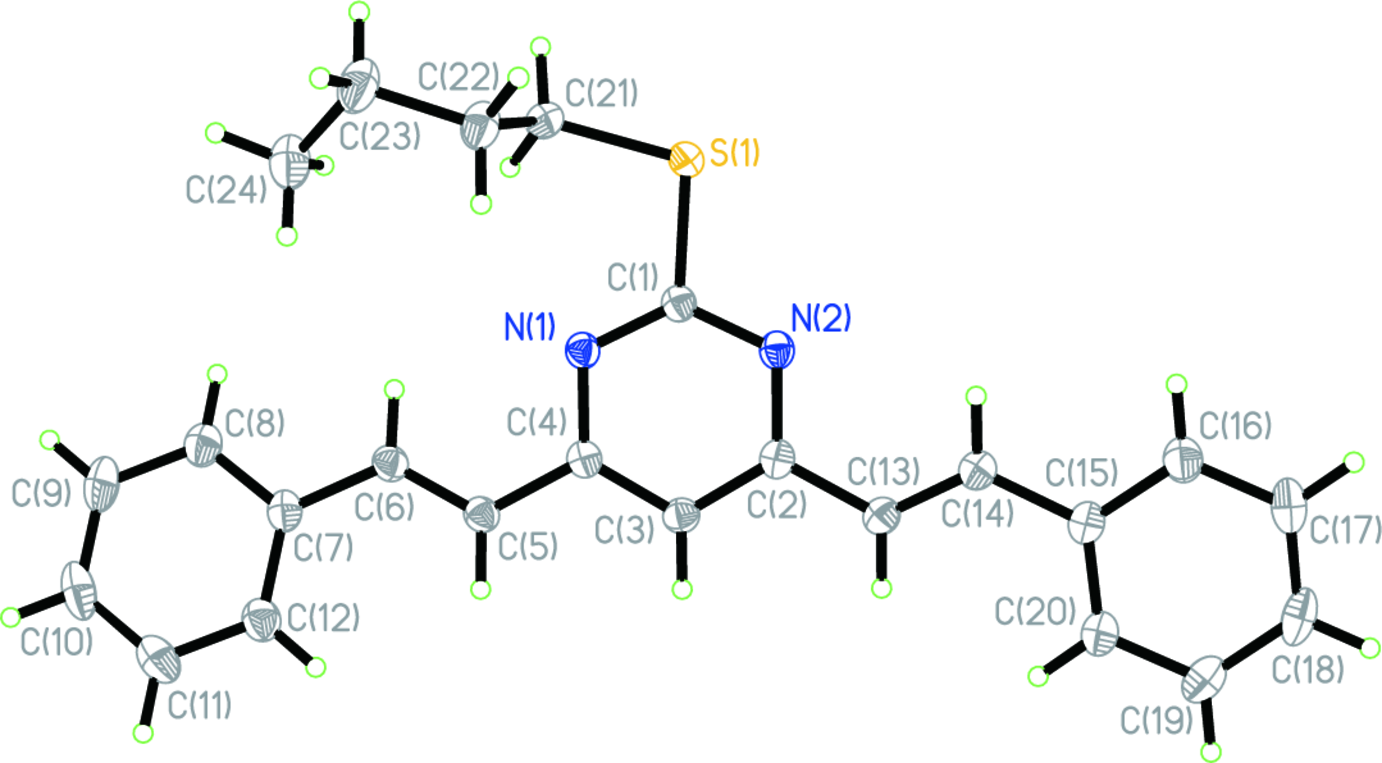

Supplement: Supplementary file 5 [file e-71-0o368-fig1.tif]

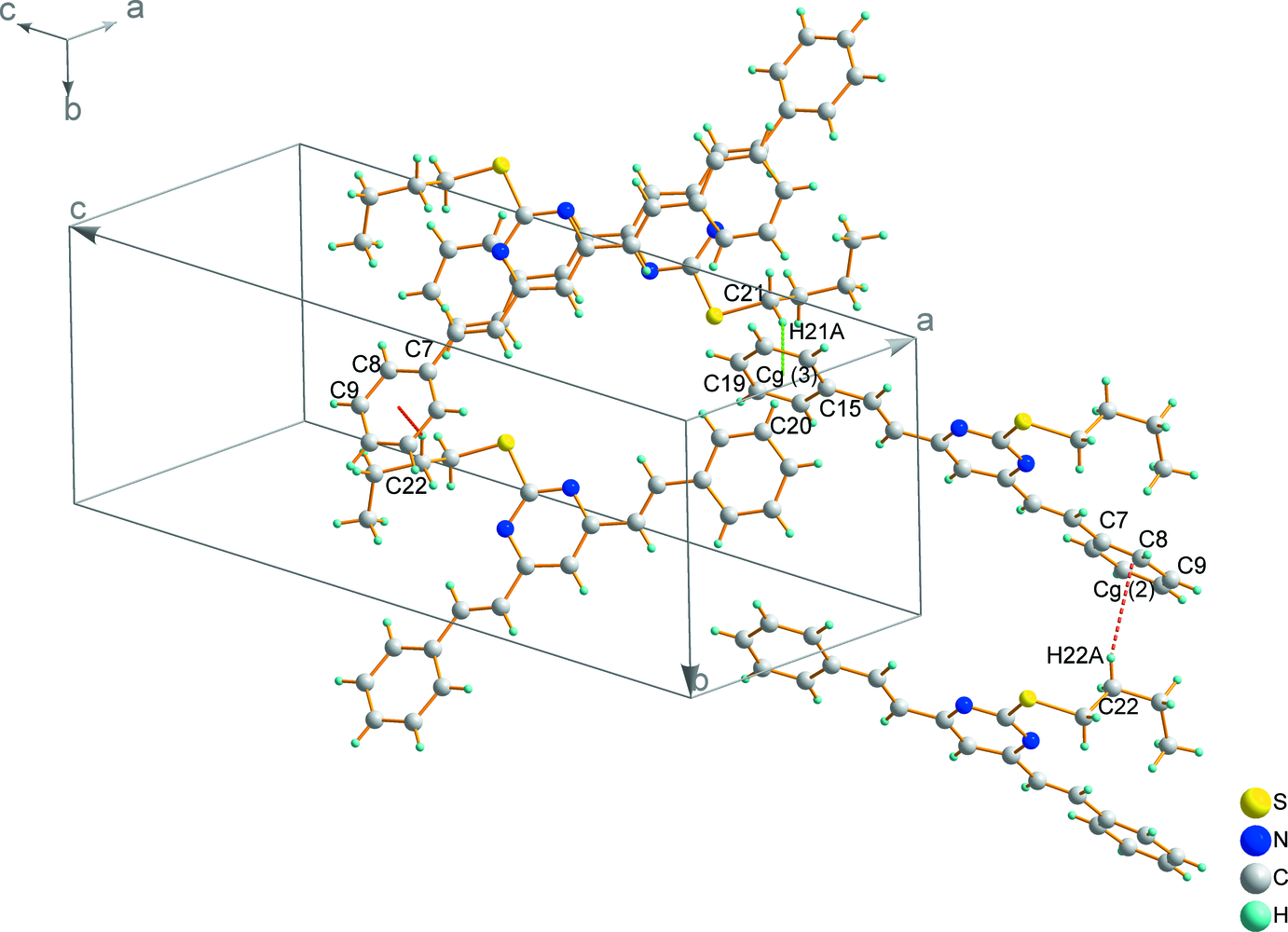

Supplement: Supplementary file 6 [file e-71-0o368-fig2.tif]
